# Supplementary material for: An in-depth analysis of data reduction methods for sustainable deep learning
Source: Open Res Eur. 2024 Sep 18;4:101. Originally published 2024 May 20. [Version 2] doi: 10.12688/openreseurope.17554.2 (PMC11413558; doi:10.12688/openreseurope.17554.2)
Supplement: Peer review history

# Reviewer report: Reviewer response for version 2

**DOI:** 10.21956/openreseurope.20049.r44381

**Published:** 2024-09-19

**Authors:** Ekaterina Merkurjev

**Affiliations:** 1 Michigan State University, East Lansing, Michigan, USA

The authors have addressed the concerns I have raised in my peer review report appropriately.

Is the study design appropriate and does the work have academic merit?

Yes

Is the work clearly and accurately presented and does it cite the current literature?

Partly

If applicable, is the statistical analysis and its interpretation appropriate?

Yes

Are all the source data underlying the results available to ensure full reproducibility?

Yes

Are the conclusions drawn adequately supported by the results?

Yes

Are sufficient details of methods and analysis provided to allow replication by others?

Yes

Reviewer Expertise:

graph-based machine learning

I confirm that I have read this submission and believe that I have an appropriate level of expertise to confirm that it is of an acceptable scientific standard.

# Reviewer report: Reviewer response for version 1

**DOI:** 10.21956/openreseurope.18970.r41935

**Published:** 2024-07-19

**Authors:** Tianzhixi Yin

**Affiliations:** 1 Pacific Northwest National Laboratory, Richland,, WA, USA

This study addresses an important and timely issue by exploring methods to reduce data for deep learning, thus conserving energy during model training. The authors introduce eight data reduction methods and evaluate their effectiveness in both tabular classification and object detection tasks using deep learning models. Notably, they employ the recently developed YOLO model for object detection, demonstrating its compatibility with the proposed reduction methods.

 However, I would appreciate further clarification on the justification for the chosen deep learning model architecture and hyperparameters in the tabular classification task. Specifically, the rationale behind selecting a 10-layer model with the given number of neurons needs to be elaborated, as classification results can be highly sensitive to these parameters. Additionally, considering the objective of reducing energy consumption, it would be beneficial to discuss the potential of employing a simpler model.

 One minor comment pertains to the clarity of Figures 9 and 10. The overlapping data points make it challenging to distinguish between the different methods.

 Overall, this paper is well-written and presents meaningful contributions. Effective data reduction methods not only save energy but also expedite the development of deep learning models.

Is the study design appropriate and does the work have academic merit?

Yes

Is the work clearly and accurately presented and does it cite the current literature?

Yes

If applicable, is the statistical analysis and its interpretation appropriate?

Yes

Are all the source data underlying the results available to ensure full reproducibility?

Yes

Are the conclusions drawn adequately supported by the results?

Yes

Are sufficient details of methods and analysis provided to allow replication by others?

Yes

Reviewer Expertise:

deep learning, statistics

I confirm that I have read this submission and believe that I have an appropriate level of expertise to confirm that it is of an acceptable scientific standard.

# Reviewer report: Reviewer response for version 1

**DOI:** 10.21956/openreseurope.18970.r41936

**Published:** 2024-07-18

**Authors:** Ekaterina Merkurjev

**Affiliations:** 1 Michigan State University, East Lansing, Michigan, USA

Dear authors,

  The work "An in-depth analysis of data reduction methods for sustainable deep learning" presents different methods for data reduction and applies them to different applications such as image data sets for object detection.

      Overall, the paper has many good aspects, such as a good experimental section, organization, etc. My main concern is the exposition- the paper needs to be edited and polished before it can be indexed. I suggest that the authors carefully edit the paper to make sure the language and exposition are up to par with the journal.

     In fact, there are many parts of the paper whose language  can be edited. For example, in the Abstract and Introduction, some words do not need to be capitalized, such as deep learning and artificial intelligence. Moreover, many sentences are awkward/incorrect to a native English speaker, such as "We also introduce a representativeness metric based on topology to measure how similar are the reduced datasets and the full training dataset." in the Abstract, and “However, notice there is no reduction method” of Section 2.3. There are also typos such as "“For example, recent publications such as 2 and 3 explain various ways to improve”. I would also not cut Algorithms 2, 6 and 8 between two pages.

     Overall, please polish the paper and go through the paper carefully to make sure the language is at the level it needs to be.

Is the study design appropriate and does the work have academic merit?

Yes

Is the work clearly and accurately presented and does it cite the current literature?

Partly

If applicable, is the statistical analysis and its interpretation appropriate?

Yes

Are all the source data underlying the results available to ensure full reproducibility?

Yes

Are the conclusions drawn adequately supported by the results?

Yes

Are sufficient details of methods and analysis provided to allow replication by others?

Yes

Reviewer Expertise:

graph-based machine learning

I confirm that I have read this submission and believe that I have an appropriate level of expertise to confirm that it is of an acceptable scientific standard, however I have significant reservations, as outlined above.
